# Supplementary material for: Content-rich biological network constructed by mining PubMed abstracts
Source: BMC Bioinformatics. 2004 Oct 8;5:147. doi: 10.1186/1471-2105-5-147 (PMC528731; doi:10.1186/1471-2105-5-147)
Supplement: Additional File 2 — The original results of the above study (non-essential files are deleted to keep the file size under the limit set by BMC bioinformatics). [file 1471-2105-5-147-S2.bz2 › chilibotAdditionalFile2/dip05/595ID9380672E10969/html/CTADII_CTAC.html]

 


 **CTADII** and **CTAC** 
  
Found 1 abstracts in PubMed, retrieved 1.  
 

 What does Google say? 
 PDF only 
| .edu only 

---

**Interactive relationship** (e.g. stimulation, inhibition, etc)

**Non-interactive relationship** (e.g. studied together, co-existance, homology, etc.)

- The  **ctaDII**  and  **ctaC**  promoters, which control the expression of the aa3 type cytochrome c oxidase subunits I and II, respectively, were not regulated by FnrP.  Ref: 11298768 Eur J Biochem, 2001
- The activities of the latter two promoters did decrease with decreasing oxygen concentrations in the growth medium, suggesting that an additional oxygen sensing mechanism exists that regulates transcription of  **ctaDII**  and  **ctaC** .  Ref: 11298768 Eur J Biochem, 2001
- ... the  **ctaDII**  and  **ctaC**  promoters did not.  Ref: 11298768 Eur J Biochem, 2001
